# Impact of a pulmonary embolism response team on the management of acute pulmonary embolism: a retrospective observational study

**DOI:** 10.1016/j.rpth.2026.106941

**Published:** 2026-09-07

**Authors:** Wei Liang Tan

**Affiliations:** Department of General Medicine, Khoo Teck Puat Hospital, National Healthcare Group, Singapore

Acute pulmonary embolism (PE) is a heterogeneous condition in which outcomes are largely determined by early risk stratification and timely initiation of appropriate therapy [1]. While anticoagulation remains the cornerstone of treatment, patients with intermediate-high- and high-risk PE may require advanced reperfusion strategies, including systemic thrombolysis and catheter-directed interventions [[2], [3], [4], [5], [6]]. The PE response team (PERT) model enables rapid multidisciplinary evaluation and risk-adapted treatment decisions, and prior studies have linked it to increased use of advanced therapies and improved care coordination [[7], [8], [9], [10]]. However, its impact on clinical outcomes and resource utilization remains variable, and data from Asian healthcare settings, where differences in healthcare structure, resource availability, and patient populations may influence PERT implementation and outcomes, remain limited.

In January 2024, a PERT service was established at Khoo Teck Puat Hospital, Singapore, as part of a broader institutional effort to standardize PE management. This study evaluates its impact by comparing treatment patterns, clinical outcomes, and resource utilization before and after implementation.

This retrospective observational study was conducted in accordance with the (Strengthening The Reporting of Observational Studies in Epidemiology) STROBE guidelines. Because this was a quality improvement audit, formal institutional review board approval was not required. Adult patients with acute PE were identified using International Classification of Diseases codes in the Epic electronic medical record system and confirmed radiologically by computed tomography pulmonary angiography. Patients were categorized into pre-PERT (January to December 2023) and post-PERT (January to December 2024) cohorts. In the post-PERT cohort, management followed a standardized institutional algorithm adapted from a previously published Singapore PERT protocol [7] and incorporating the 2019 European Society of Cardiology (ESC) risk stratification framework [1]. PERT included a multidisciplinary team of general medicine, cardiology, respiratory medicine, interventional radiology, and critical care specialists, enabling real-time case discussion and individualized treatment decisions.

Patients were classified using the 2019 ESC risk stratification framework, applied retrospectively with identical criteria across both cohorts. During retrospective case review, right ventricular size and cardiac biomarker data were examined prior to formal Pulmonary Embolism Severity Index calculation, which differs from the hierarchical sequence specified in the ESC algorithm. Final risk categories were assigned by reconciling all available clinical, imaging, and biochemical data, though the possibility of misclassification at risk-tier boundaries cannot be excluded.

Primary outcomes were advanced therapy utilization and hospital length of stay (LOS). Secondary outcomes included intensive care unit (ICU)/high-dependency unit admission, survival to discharge, bleeding complications (defined by the International Society on Thrombosis and Haemostasis criteria [11]), 30-day venous thromboembolism recurrence, and 30-day mortality. A multivariable linear regression model, adjusted for age and baseline PE risk stratification, assessed the independent association between PERT implementation and hospital LOS. A prespecified subgroup analysis compared outcomes by treatment modality among intermediate-high- and high-risk patients; patients receiving combined therapies or no anticoagulation were excluded. Given the small sample size, subgroup findings are considered exploratory.

A total of 192 patients were included: 126 pre-PERT and 66 post-PERT. The smaller post-PERT cohort reflects a redistribution of the catchment population following the opening of a new regional hospital in 2024. Baseline demographics were similar between groups. The post-PERT cohort had a higher proportion of intermediate-high-risk PE (43.9% vs 19.1%), while high-risk PE was comparable (7.6% vs 9.5%). Because stratification was applied retrospectively using identical criteria, this difference reflects a change in case mix rather than improved risk recognition. Demographics and risk profiles are summarized in Table 1.

**Table 1:** Patient demographics, risk profiles, treatment patterns, and clinical outcomes.

Variable	Pre-PERT (2023) *n* = 126	Post-PERT (2024) *n* = 66	*P* value
Demographics			
Age (y), mean ± SD	66.4 ± 16.5	67.5 ± 15.8	
Male, %	41.3	43.9	
BMI (kg/m^2^), mean ± SD	25.2 ± 8.6	26.6 ± 7.5	
Risk stratification (ESC 2019), %			
Low	34.1	24.2	
Intermediate-low	37.3	24.2	
Intermediate-high	19.1	43.9	
High	9.5	7.6	
Treatment patterns, %			
Anticoagulation	85.7	89.4	.47
Apixaban	49.2	65.2	
Warfarin	12.7	1.5	
**Advanced interventions**	**19.0**	**31.8**	**.047**
Systemic thrombolysis	5.6	13.6	.05
CDT	5.6	13.6	.05
IVC filter insertion	8.7	12.1	
Clinical outcomes			
**Hospital LOS (d), median (IQR)**	**12.5 (7.0-23.0)**	**9.0 (6.0-16.5)**	**.02**
**Adjusted reduction in LOS (95% CI)**	**-**	**−4.5 (−8.88 to −0.17)**	**.04**
Admission to HD/ICU, %	30.2	34.8	.51
Survival to discharge, %	84.9	87.9	.97
In-hospital bleeding complications, %	18.3	12.1	.31
30-d VTE recurrence, %	2.4	0	.55
30-d mortality, %	4.8	7.6	.52

Bolded values indicate statistical significance (*P* < .05).

BMI, body mass index; CDT, catheter-directed thrombectomy; ESC, European Society of Cardiology; HD, high dependency; ICU, intensive care unit; IVC, inferior vena cava; LOS, length of stay; PERT, pulmonary embolism response team; VTE, venous thromboembolism.

PERT implementation was associated with increased use of direct oral anticoagulants, particularly apixaban (65.2% vs 49.2%), and a marked reduction in warfarin use (1.5% vs 12.7%). Utilization of advanced therapies increased (31.8% vs 19.0%; *P* = .047), predominantly among intermediate-high- and high-risk patients. Despite the higher risk profile, median hospital LOS was shorter in the post-PERT cohort (9.0 days; IQR, 6.0-16.5 vs 12.5 days; IQR, 7.0-23.0; *P* = .02), and this association persisted in multivariable analysis (adjusted reduction, 4.5 days; 95% CI, −8.88 to −0.17; *P* = .04). This is consistent with a 2025 meta-analysis [12] of 14 studies (*N* = 12,337) reporting a mean hospital LOS reduction of 1.49 days with PERT, and with the 2026 American Heart Association/American College of Cardiology PE guideline's [13] observation that PERT is associated with reduced hospital and ICU LOS in most studies. There were no significant differences in ICU/high-dependency admission rates, survival to discharge, bleeding complications, 30-day venous thromboembolism recurrence, or 30-day mortality. Treatment patterns and outcomes are presented in Table 1.

In subgroup analyses of intermediate-high- and high-risk patients, survival differed across treatment modalities (*P* = .046), with the highest survival in the catheter-directed thrombectomy (CDT) group (92.3%), followed by anticoagulation alone (81.1%) and systemic thrombolysis (64.3%). Bleeding complications and clinical deterioration were numerically lower in the CDT group but did not reach statistical significance. Subgroup outcomes are presented in Table 2.

**Table 2:** Subgroup analysis: outcomes for intermediate-high- and high-risk pulmonary embolism by treatment modality.

Outcome	Anticoagulation alone (*n* = 37), %	Systemic thrombolysis (*n* = 14), %	CDT (*n* = 13), %	*P* value
**Hospital LOS (d), median (IQR)**	**11.0 (7.0-22.0)**	**6.0 (3.8-9.5)**	**12.0 (8.5-24.5)**	**.006**
**Admission to HD/ICU**	**45.9**	**100**	**100**	**<.001**
**Survival to discharge**	**81.1**	**64.3**	**92.3**	**.046**
Bleeding complications	10.8	14.3	23.1	.42
Clinical deterioration	29.7	35.7	7.6	.21
Cardiac arrest	21.6	35.7	7.6	.22
30-d mortality	8.1	0	0	.57

Bolded values indicate statistical significance (*P* < .05).

CDT, catheter-directed thrombectomy; HD, high dependency; ICU, intensive care unit; LOS, length of stay.

PERT implementation was associated with a shift toward contemporary anticoagulation practice and increased utilization of advanced therapies. Despite a higher-risk case mix, key clinical outcomes, including ICU utilization, bleeding complications, and mortality, remained unchanged, suggesting that a more aggressive, risk-adapted approach can be implemented safely within a structured multidisciplinary framework. The reduction in hospital LOS likely reflects improved care coordination and earlier decision-making, though concurrent healthcare system restructuring is an important cointervention that limits attribution of this effect solely to PERT.

The survival signal favoring CDT in the subgroup analyses is hypothesis-generating only. Given the small sample size, nonrandomized design, and likely confounding by indication, causal inference is not warranted. These observations are consistent with emerging evidence from the (Evaluating the Safety and Efficacy of the Indigo aspiration system in Acute Pulmonary Embolism) EXTRACT-PE and (FlowTriever Pulmonary Embolectomy Clinical Study) FLARE trials supporting catheter-based strategies in selected patients [[4], [5], [6]] and warrant prospective evaluation.

This study contributes to the limited evidence base on Asian healthcare systems. The retrospective, single-center design, small sample size, and 1-year observation window limit the strength of the conclusions. Residual confounding cannot be excluded despite multivariable adjustment. Additionally, retrospective risk stratification introduced a sequencing deviation from the ESC algorithm, in which right ventricular size and biomarker data were reviewed prior to Pulmonary Embolism Severity Index calculation; the possibility of misclassification at risk-tier boundaries cannot be fully excluded. Future work should incorporate longer observation periods, multicenter data, and prospective designs to evaluate PERT more rigorously in Asian healthcare settings.

In conclusion, PERT implementation at a public hospital in Singapore was associated with increased utilization of advanced therapies, a shift toward contemporary anticoagulation practices, and reduced hospital LOS, independent of baseline risk profile. These findings support the value of structured multidisciplinary care models for optimizing PE management in Asian healthcare systems, and the observed survival signal with CDT in higher-risk patients warrants further prospective investigation.
